# Polyclonal Hypergammaglobulinemia in Severe Hidradenitis Suppurativa

**DOI:** 10.7759/cureus.100711

**Published:** 2026-01-03

**Authors:** Cristina P Gerena-Maldonado, Mariana Sadurní-García, Gerardo S Caussade-Silvestrini, Coral Martes-Villalobos, Alma Cruz

**Affiliations:** 1 Department of Dermatology, University of Puerto Rico, Medical Sciences Campus, San Juan, USA; 2 Department of Dermatology, University of Puerto Rico, Medical Sciences Campus, School of Medicine, San Juan, USA; 3 Department of Dermatology, Universidad Central del Caribe, School of Medicine, Bayamón, USA

**Keywords:** biomarkers, hidradenitis suppurativa, infliximab, polyclonal hypergammaglobulinemia, serum immunoglobulin

## Abstract

This is the case of a 36-year-old man with a 15-year history of Hurley stage III hidradenitis suppurativa (HS) who presented with painful nodules, chronic drainage, and extensive sinus tracts involving the axillae, groin, and gluteal cleft. The patient had previously failed multiple systemic and procedural therapies, including oral antibiotics, intralesional corticosteroids, adalimumab, and methotrexate. Physical examination demonstrated hypertrophic scarring, purulent discharge, and diffuse inflammatory activity. Laboratory evaluation revealed marked polyclonal hypergammaglobulinemia (pHGG), a finding whose association with HS is increasingly reported but remains incompletely understood. Therefore, we present an interesting case of severe, refractory HS with pHGG that improved after infliximab treatment, along with a brief discussion of relevant literature.

## Introduction

Hidradenitis suppurativa (HS) is a chronic, relapsing inflammatory disorder of hair follicles with systemic manifestations. Hematologic abnormalities such as neutrophilia, reactive thrombocytosis, and anemia of inflammation are recognized; however, polyclonal hypergammaglobulinemia (pHGG) is less emphasized despite increasing reports in the literature [1-3]. pHGG likely reflects widespread B‑cell activation due to chronic inflammation rather than clonal plasma‑cell disease. A recent cohort study demonstrated a high prevalence of hypergammaglobulinemia in HS [3]. Prevalence rates exceeded 60% overall and approached 80% among individuals aged 15-29 years [3]. Additionally, patients with severe HS in this cohort, especially in younger age groups, showed a diminished clinical response to adalimumab [3]. We present a case of severe, refractory HS with pHGG that improved after infliximab therapy, along with a brief discussion of the pertinent literature.

## Case presentation

**Figure 1:**
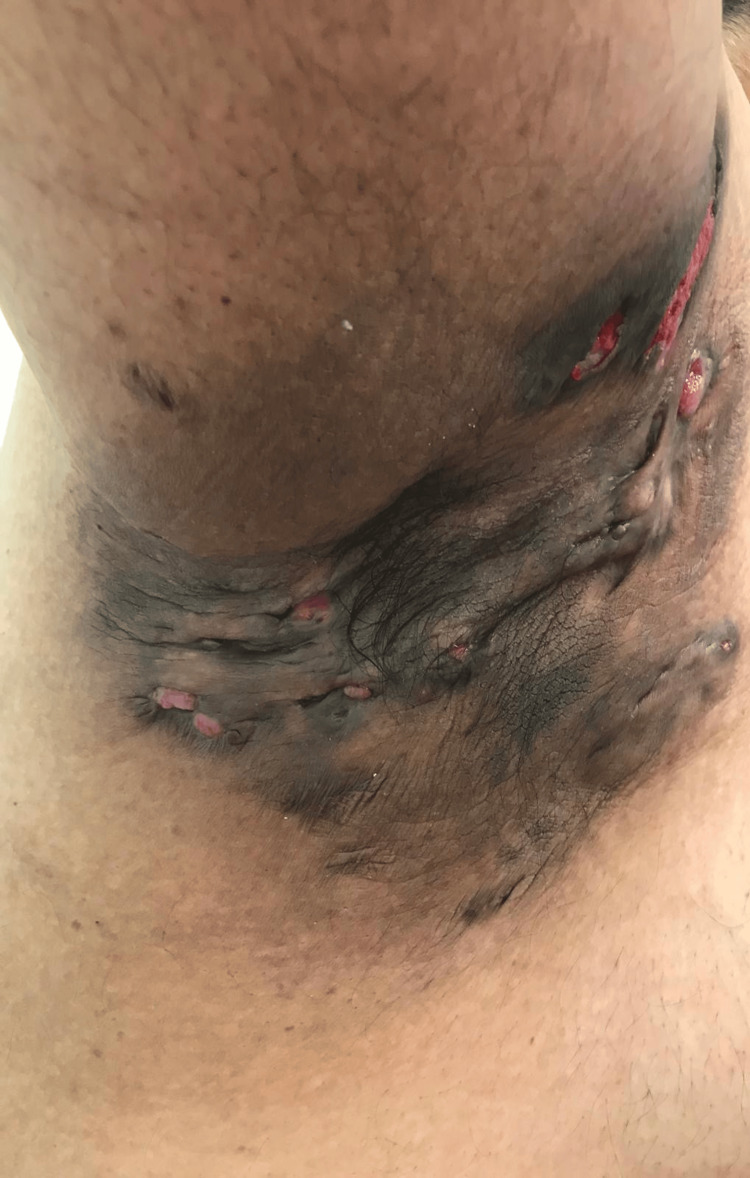
Hurley stage III disease of the right axilla with open HS lesions and interconnected sinus tracts HS: Hidradenitis suppurativa.

**Figure 2:**
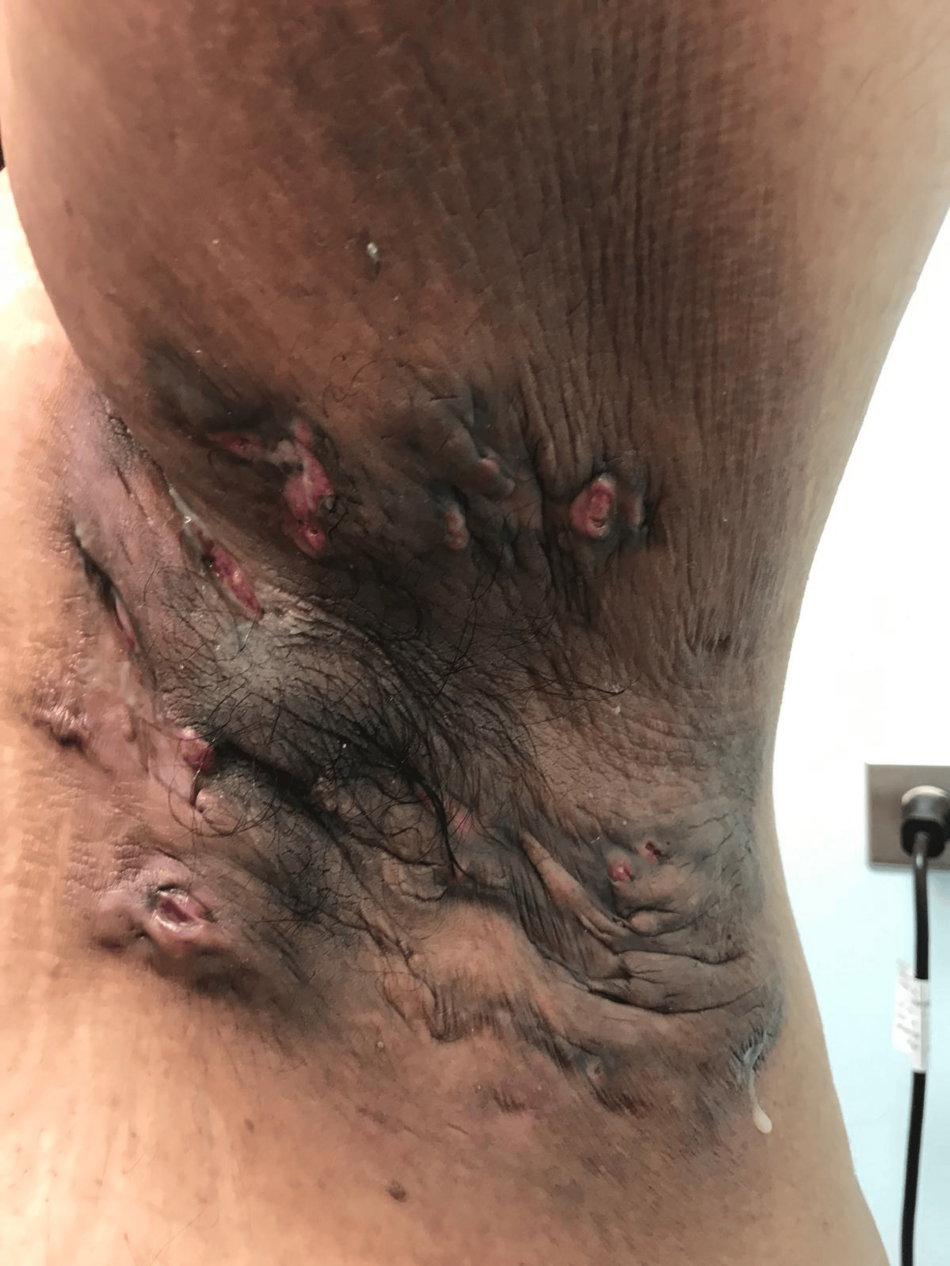
Hurley stage III disease of the left axilla, marked by draining and interconnected sinus tracts

A 36-year-old man with a 15-year history of HS, who is an active smoker, presented with painful, draining lesions across both axillae, groin, and the gluteal cleft. The axillary draining tracts, illustrated in Figures 1, 2, reflected Hurley stage III disease [4] with cicatricial bands and interconnected sinus tracts.

The patient previously received several treatments, including oral antibiotics, intralesional corticosteroids, adalimumab, and methotrexate. Adalimumab was administered at standard induction and maintenance doses: 160 mg on day zero, 80 mg at week two, and then 40 mg each week. The patient was also treated with oral methotrexate, titrated to a maximum dose of 15 mg weekly for several months. None of these treatments led to disease control or improvement in associated hematologic laboratory abnormalities. Criteria for treatment failure, such as International Hidradenitis Suppurativa Severity Score System (IHS4) or Hidradenitis Suppurativa Clinical Response (HiSCR) were not reported.

Over more than 12 months, serial complete blood counts (CBCs), as summarized in Table 1, demonstrated neutrophilic leukocytosis (peak 18.0×10³/µL), microcytic anemia (hemoglobin 10.3-11.5 g/dL; Mean Corpuscular Volume (MCV) ~75 fL), and thrombocytosis (>500×10³/µL).

**Table 1:** Temporal progression of CBC abnormalities and relevant laboratory findings CBC, complete blood count; WBC, white blood cell count; Hgb, hemoglobin; g/dL, grams per deciliter; MCV, mean corpuscular volume; Hct, hematocrit; CMP, comprehensive metabolic panel; fL, femtoliters; µL, microliter.

Date	WBC (×10³/µL); Reference range: 4.5-11.0	Hgb (g/dL); Reference range: 14.0-18.0	Platelets (×10³/µL); Reference range: 150-425	Additional findings
Oct 19, 2023	14.7	11.0	510	MCV 77.0 fL; CMP within normal limits
Feb 5, 2024	18.0	10.3	>500	Hct 33.5%, MCV 75.3 fL, Neutrophils 77%; CMP within normal limits
Aug 19, 2024	13.7	10.4	>500	CMP within normal limits
Oct 21, 2024	12.4	11.5	>500	CMP within normal limits

The metabolic panels, also shown in Table 1, remained within normal limits throughout this period. Serum protein electrophoresis with immunofixation revealed IgG 2953 mg/dL; with a polyclonal pattern and no monoclonal spike. Tests for myeloproliferative disorders (JAK2 V617F, CALR, MPL) and BCR‑ABL were negative. Screening for human immunodeficiency virus (HIV), hepatitis B, and latent tuberculosis (TB) was negative. There was no lymphadenopathy, hepatosplenomegaly, or B‑symptoms.

Given the refractory nature and systemic inflammation, infliximab was started at 5 mg/kg at weeks zero, two, and six, then every eight weeks. Within months, inflammatory signs and drainage decreased significantly. Additionally, hematologic abnormalities improved, including decreased leukocytosis and thrombocytosis, and increased hemoglobin levels. Repeat laboratory testing demonstrated serum IgG levels below 1,500 mg/dL (reference range: 700-1,600 mg/dL), although the precise quantitative value was not available in the medical record. Furthermore, serial measurements by the hematologist/oncologist demonstrated a consistent downward trend in serum IgG levels. Follow-up serum protein electrophoresis could not be performed due to lack of health insurance coverage. The patient remains on maintenance therapy with sustained clinical improvement.

## Discussion

HS involves dysregulated innate and adaptive immunity, with elevated levels of cytokines such as interleukin (IL)‑1β, IL‑17, tumor necrosis factor (TNF)‑α, and IL‑6 [5-7]. IL‑6 prompts hepcidin production (causing iron-restricted erythropoiesis), induces neutrophilia, and enhances thrombopoiesis, explaining the hematologic triad observed in this patient [8,9]. In this case, the patient's anemia was mainly seen as a sign of chronic inflammatory disease, rather than a standalone hematologic problem. Previous studies have found a strong association between HS and anemia, consistent with the immune dysregulation observed in severe cases [10]. Furthermore, chronic B‑cell stimulation in this inflammatory environment can lead to polyclonal increases in immunoglobulin levels [1,11].

While pHGG is not commonly highlighted in HS, several reports support its association with disease activity. A research study noted pHGG with elevated acute‑phase reactants, suggesting a reactive process [1]. A pediatric case demonstrated improvement in gammopathy with better HS control [2]. A single-center cohort associated hypergammaglobulinemia with more severe disease and lower response rates to adalimumab [3]. Additional reports showed IgG subclass perturbations (e.g., IgG4) in severe HS, which may risk misdiagnosis as IgG4‑related disease [12]. Furthermore, biomarker studies indicate that total IgG correlates with HS severity indices [11].

The clinical implications of these findings are that when globulins or total Ig are elevated in HS, clinicians should undertake (i) serum protein electrophoresis with immunofixation to distinguish polyclonal from monoclonal patterns, (ii) an evaluation for infection and systemic inflammation, and (iii) optimization of HS control, as improved disease management may help reduce pHGG. In patients with prior adalimumab exposure and inadequate control, escalation or class switch to infliximab can be considered for Hurley III disease, as in our patient, with interval laboratory reassessment to document hematologic and immunologic response. In this case, infliximab controlled HS disease activity better than adalimumab. Although conclusions cannot be drawn from a single case, earlier studies have shown more clinical and inflammatory improvement with infliximab than with standard-dose adalimumab in patients with severe or hard-to-treat HS [13]. Additional biologic agents targeting the IL-17 pathway, including bimekizumab and secukinumab, have been evaluated in the treatment of HS [14]. Some clinical studies have identified secukinumab as a safe option for patients who exhibit an inadequate response or failure to adalimumab [15]. In the absence of red flags for hematologic malignancy (monoclonality, cytopenias, organomegaly, constitutional symptoms), a treatment-first approach with follow-up laboratories can be reasonable and an unnecessary bone marrow biopsy may be averted [1,2].

This report involves a single case; thus, causality cannot be confirmed. Existing literature is limited and heterogeneous, with sparse longitudinal immunoglobulin data. Further studies should also focus on determining whether pHGG is a marker associated with the disease or whether it causally contributes to HS pathogenesis. It is important to note that although pHGG has been documented in HS, evidence linking HS to monoclonal hypergammaglobulinemia remains even more limited. Understanding the association between pHGG and HS could lead to a diagnostic/prognostic tool to detect HS at earlier stages or to identify at-risk individuals.

## Conclusions

This case illustrates that pHGG in HS may represent a reactive process driven by chronic inflammation rather than a clonal disorder. Recognizing this association may guide appropriate laboratory evaluation and prevent unnecessary invasive testing. Effective HS management, particularly with biologic therapies such as infliximab, may result in the improvement of associated hematologic disturbances, including pHGG. However, the reduction in pHGG should not be interpreted as a direct consequence of infliximab therapy alone, as it may reflect adequate disease management. Additional studies are warranted to determine the prevalence and significance of pHGG in HS.
